# Neurological Symptom Improvement After Re-Irradiation in Patients With Diffuse Intrinsic Pontine Glioma: A Retrospective Analysis of the SIOP-E-HGG/DIPG Project

**DOI:** 10.3389/fonc.2022.926196

**Published:** 2022-06-22

**Authors:** Lara Chavaz, Geert O. Janssens, Stephanie Bolle, Henry Mandeville, Monica Ramos-Albiac, Karen Van Beek, Helen Benghiat, Bianca Hoeben, Andres Morales La Madrid, Clemens Seidel, Rolf-Dieter Kortmann, Darren Hargrave, Lorenza Gandola, Emilia Pecori, Dannis G. van Vuurden, Veronica Biassoni, Maura Massimino, Christof M. Kramm, Andre O. von Bueren

**Affiliations:** ^1^ Department of Pediatrics, Gynecology and Obstetrics, Division of Pediatric Hematology and Oncology, University Hospital of Geneva, Geneva, Switzerland; ^2^ Cansearch Research Platform for Pediatric Oncology and Hematology, Faculty of Medicine, Department of Pediatrics, Gynecology and Obstetrics, University of Geneva, Geneva, Switzerland; ^3^ Department of Radiation Oncology, University Medical Center Utrecht, Utrecht, Netherlands; ^4^ Princess Maxima Center for Pediatric Oncology, Utrecht, Netherlands; ^5^ Department of Radiation Oncology, Gustave Roussy, Paris Saclay University, Villejuif, France; ^6^ Department of Radiotherapy, The Royal Marsden Hospital and Institute of Cancer Research, Sutton, United Kingdom; ^7^ Department of Radiation Oncology, Hospital Vall d’Hebron, Barcelona, Spain; ^8^ Department of Radiation Oncology, University Hospitals Leuven, Leuven, Belgium; ^9^ Department of Clinical Oncology, University Hospital Birmingham, Birmingham, United Kingdom; ^10^ Department of Pediatric Oncology, Hospital Sant Joan de Deu, Barcelona, Spain; ^11^ Department of Radiation-Oncology, University Hospital Leipzig, Leipzig, Germany; ^12^ Pediatric Oncology Unit, Great Ormond Street Hospital for Children, London, United Kingdom; ^13^ Pediatric Radiotherapy Unit, Fondazione Istituto di Ricovero e Cura a Carattere Scientifico (IRCCS) Istituto Nazionale dei Tumori, Milan, Italy; ^14^ Department of Pediatric Oncology, Emma Children’s Hospital, Amsterdam University Medical Centers (UMC), Vrije Universiteit Amsterdam, Amsterdam, Netherlands; ^15^ Pediatrics Unit, Fondazione Istituto di Ricovero e Cura a Carattere Scientifico (IRCCS) Istituto Nazionale dei Tumori, Milan, Italy; ^16^ Division of Pediatric Hematology and Oncology, University Medical Center Goettingen, Goettingen, Germany

**Keywords:** diffuse intrinsic pontine glioma (DIPG), radiotherapy, re-irradiation (re-RT), child, adolescent

## Abstract

**Purpose:**

The aim of this study is to investigate the spectrum of neurological triad improvement in patients with diffuse intrinsic pontine glioma (DIPG) treated by re-irradiation (re-RT) at first progression.

**Methods:**

We carried out a re-analysis of the SIOP-E retrospective DIPG cohort by investigating the clinical benefits after re-RT with a focus on the neurological triad (cranial nerve deficits, ataxia, and long tract signs). Patients were categorized as “responding” or “non-responding” to re-RT. To assess the interdependence between patients’ characteristics and clinical benefits, we used a chi-square or Fisher’s exact test. Survival according to clinical response to re-RT was calculated by the Kaplan–Meier method.

**Results:**

As earlier reported, 77% (*n* = 24/31) of patients had any clinical benefit after re-RT. Among 25/31 well-documented patients, 44% (*n* = 11/25) had improvement in cranial nerve palsies, 40% (*n* = 10/25) had improvement in long-tract signs, and 44% (11/25) had improvement in cerebellar signs. Clinical benefits were observed in at least 1, 2, or 3 out of 3 symptoms of the DIPG triad, in 64%, 40%, and 24%, respectively. Patients irradiated with a dose ≥20 Gy versus <20 Gy may improve slightly better with regard to ataxia (67% versus 23%; *p*-value = 0.028). The survival from the start of re-RT to death was not different between responding and non-responding DIPG patients (*p*-value = 0.871).

**Conclusion:**

A median re-irradiation dose of 20 Gy provides a neurological benefit in two-thirds of patients with an improvement of at least one symptom of the triad. DIPG patients receiving ≥20 Gy appear to improve slightly better with regard to ataxia; however, we need more data to determine whether dose escalation up to 30 Gy provides additional benefits.

## Introduction

Radiotherapy (RT) is still considered as the cornerstone of treatment for patients with diffuse intrinsic pontine glioma (DIPG) (1–4). Current standard of care for newly diagnosed DIPG patients includes at least focal RT up to a dose of 54 Gy, or a low-burden alternative up to 39 Gy (1, 5). Tinkle et al. evaluated the evolution of neurological triad symptoms of patients with DIPG undergoing upfront RT. Among 108 patients, 57% of the patients had the classical triad (6), composed of cerebellar signs (CB) (e.g., ataxia, dysmetria, and dysarthria), cranial neuropathy (CN) (e.g., diplopia), and long-tract (LT) signs (e.g., paraplegia, Babinski sign, and hyperreflexia) at diagnosis. Neurological improvement in CB, CN, and LT signs at the end of upfront RT is 87.9%, 85.4%, and 80.8%, respectively (2).

Numerous studies have investigated the impact of re-RT in the management of DIPG at first progression, in particular by studying the effect on overall survival (OS) (7–14). To our knowledge, the SIOP-E (Société International d’Oncologie Pédiatrique) working group (7) reported on the largest retrospective matched cohort comparing patients with DIPG undergoing re-RT (*n* = 31) to DIPG patients who did not receive a second course of RT (*n* = 39). We concluded that a significant benefit in median OS (13.7 versus 10.3 months; *p*-value = 0.04) was observed in favor of re-irradiated children. Moreover, the global clinical improvement after re-RT was documented in 77% of patients (7). Similar frequencies were reported to be ranging from 67% (10) to 100% (13) across other studies (15).

However, to date, studies investigating the prevalence and spectrum of the typical triad symptom changes after re-RT in progressive DIPG patients are missing. We also aimed to better understand whether certain variables may increase the probability to respond to re-RT. In addition, we investigated whether DIPG patients showing a clinical benefit to re-RT are characterized by a different survival.

## Materials and Methods

### Eligibility

This study is a re-analysis of the SIOP-E retrospective cohort published before (7). Similar eligible and exclusion criteria are used. Patients aged <18 years and fulfilling the following criteria were eligible: (1) clinical and radiological signs of a typical DIPG at initial diagnosis or a biopsy confirming high-grade glioma, and (2) an interval of ≥3 months between the last day of upfront RT and the start of re-RT. The combination of re-irradiation and systemic agents was allowed. Patients with leptomeningeal dissemination or multifocal disease on MRI at first progression, patients undergoing more than one course of re-irradiation, patients with systemic therapy as the third-line treatment, and children with a history of neurofibromatosis were excluded from the analysis. Between August 2011 and May 2015, a total of 31 patients with DIPG responding to the upfront RT and undergoing re-RT at first progression were included (7).

### Radiotherapy

The upfront RT and re-RT of the DIPG patients have been described elsewhere (7). In brief, conventional (dose/fraction: 1.8–2.0 Gy) and hypo-fractionated (dose/fraction: >2.0 Gy) RT regimens were permitted during upfront RT. At first progression, a re-RT regimen with a total dose between 18 Gy and 30 Gy with at least ten fractions was required for analysis.

### Responding and Non-Responding Patients

To evaluate the effect of the clinical response on survival outcomes and to assess what kind of variables may increase the probability to show a response to re-RT, we grouped the patients according to their clinical response to re-RT. Patients were categorized as “responding” or “non-responding” to treatment according to whether they showed clinical benefit post re-RT (responding, *n* = 24; non-responding, *n* = 7). In our retrospective study, not all signs of the neurological triad were systematically described in patient charts. Any documented symptom improvement (detailed or general) by the treating physician was interpreted and linked to the triad. Improvement in ataxia was frequently assessed by the Brief Ataxia Rating Scale (16).

### Objectives

The primary objective of this study was to quantify and specify the type of clinical benefits post re-RT with the focus on the neurological symptoms of the DIPG triad. The secondary objective was to assess the survival time, defined as time from re-RT to last follow-up or death, in responding and non-responding DIPG patients to re-RT. In addition, variables that may increase the probability to show a response to re-RT were analyzed.

### Statistical Analysis

All statistical analyses including descriptive statistics were done using IBM SPSS Statistics for Windows, version 26 (IBM Corp., Armonk, New York).

The impact of three variables—gender, age by group (patients were dichotomized by the median age into ≤6 years versus >6 years), and total dose of re-RT (patients were dichotomized by the median re-RT dose applied into <20 Gy versus ≥20 Gy)—on clinical benefits was investigated. To assess the interdependence between these variables and clinical benefits, either a Chi-square test or Fisher’s exact test was used.

The distribution of continuous variables like patients’ age, time interval from upfront RT to re-RT (from the last day of RT to the first day of re-RT), or progression-free survival (PFS) was not normal (Shapiro–Wilk test *p*-value <0.05); thus, means were compared using a Mann–Whitney *U* test.

Survival time was compared between responding and non-responding patients. The probability of time to death according to the clinical benefits was calculated by the Kaplan–Meier method and compared between groups using the log-rank test.

Results were described in terms of median, mean, standard deviation (SD), and percentages. A *p*-value <0.05 was considered significant. No correction for multiple testing was performed.

## Results

### Patients’ Characteristics and Treatment

**Figure 1:**
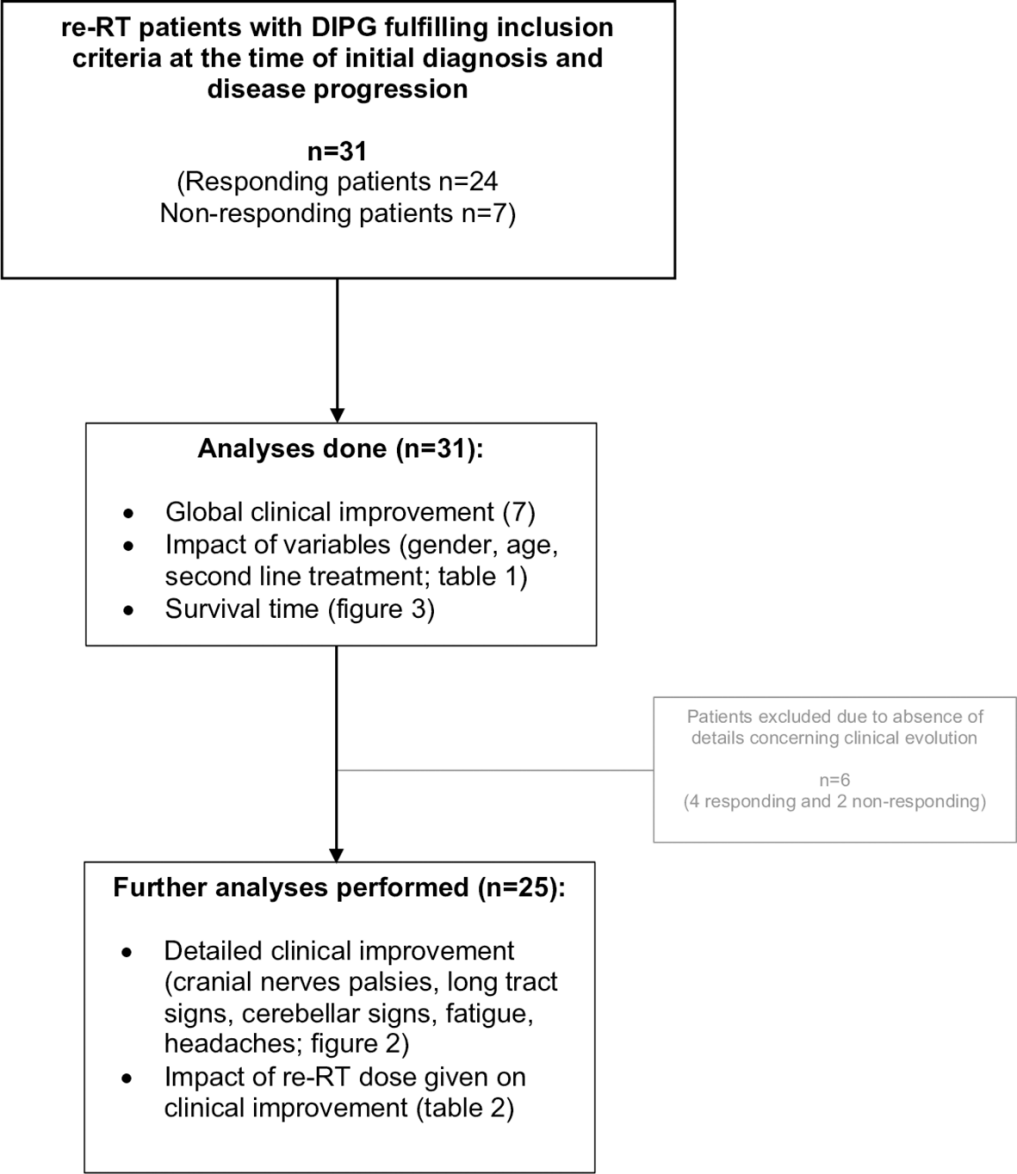
Study diagram and analyses performed.

**Figure 2:**
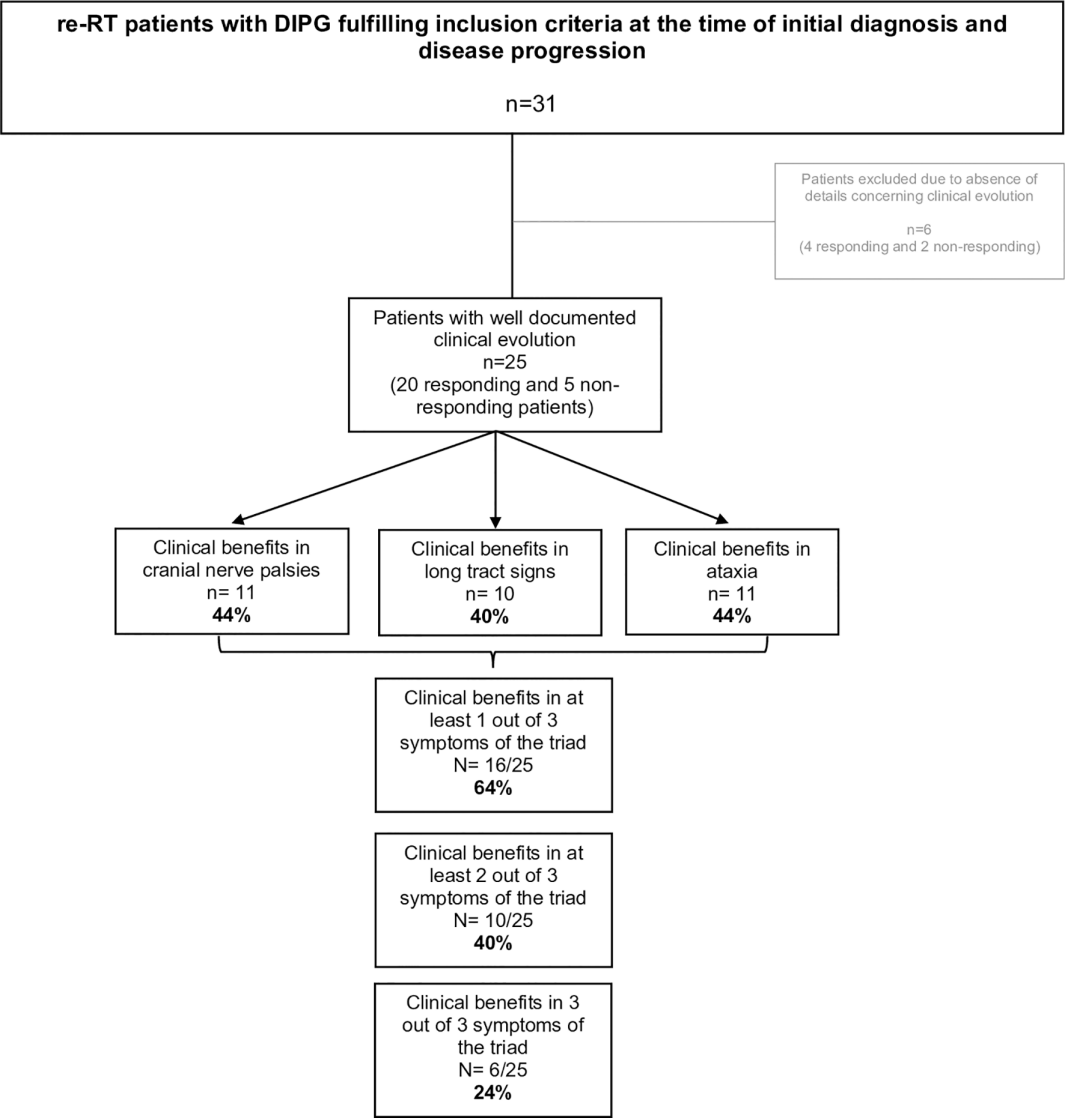
DIPG triad symptom evolution after re-RT.

Thirty-one patients were retrospectively evaluated for neurological triad symptom improvement after re-RT, 24/31 (77%) had global clinical benefits after re-RT as reported before (7) and were considered as “responding” patients, whereas 7/31 (23%) had no clinical benefits documented after re-RT and were considered as “non-responding” patients. Detailed clinical information, particularly neurological symptom improvement, was documented for 25/31 patients (20 responding and 5 non-responding) as shown in **Figures 1**, **2**. All patient characteristics are described in **Table 1**.

**Table 1:** Demographic and disease characteristics of patients grouped according to the clinical response to re-RT.

Characteristics			Responding (*n* = 24)				Non-responding (*n* = 7)				All patients (*n* = 31)			
**Patient characteristics**														
Gender														
		Male	11			46%	3			43%	14			45%
		Female	13			54%	4			57%	17			56%
Age (years)														
		Median	8				6				6			
		Range	3 to 16				2 to 13				2 to 16			
		≤6 yo	11			46%	5			71%	16			52%
		>6 yo	13			54%	2			29%	15			48%
PFS (days)														
		Median	245,5				236				245			
		Range	112 to 374				168 to 952				112 to 952			
Interval RT to re-RT (days)														
		Median	225.5				209				217			
		Range	103 to 363				112 to 930				103 to 930			
Survival time (days)														
		Median	150				90				146			
		Range	67 to 325				21 to 297				21 to 325			
**Treatment characteristics**														
Second line treatment														
		RT	12			50%	4			43%	16			52%
		RT + chemo	12			50%	3			57%	15			48%
re-RT dose given (Gy)														
		Median	20				19,8				20			
		Range	18 to 30				18 to 30				18 to 30			
		< 20 Gy	10			42%	4			57%	14			45%
		≥ 20 Gy	14			58%	3			43%	17			55%

PFS, progression-free survival. yo, years old. Interval RT to re-RT, interval of time from the last day of RT and the first day of re-RT. Survival time, survival from start of re-RT to death/last follow-up. Chemo, chemotherapy (part of the second-line treatment in addition to re-RT: nimotuzumab–vinorelbine based, n = 9; etoposide, n = 1; sirolimus, n = 2; valproic acid + celecoxib, n = 1; valproic acid + temsirolimus + irinotecan, n = 1; bevacizumab, n = 1).

Patient characteristics of children responding to re-RT were similar when compared to DIPG patients without documented clinical benefit to re-RT (**Table 1**). Neither difference in median time interval from the last day of RT to the start of re-RT (225 days versus 209 days, Mann–Whitney *U* test *p*-value = 0.839), nor difference in the time interval between diagnosis to progression (245 days versus 236 days, Mann–Whitney *U* test *p*-value = 0.321), defined as PFS, was observed.

### Rate of Neurological Triad Symptom Change After Re-RT

All patients had neurological signs at the onset of re-RT as reported previously (7). Analysis of the improvement in the triad symptoms, performed over 25 patients (20 responding and 5 non-responding), demonstrated that 16/25 (64%) patients had clinical benefits in at least one out of three symptoms of the triad, 10/25 (40%) in at least 2, and 6/25 (24%) improved in all three domains of the triad (**Figure 2**).

### Evaluation of the Survival Outcomes According to the Patients’ Response to Re-RT

**Figure 3:**
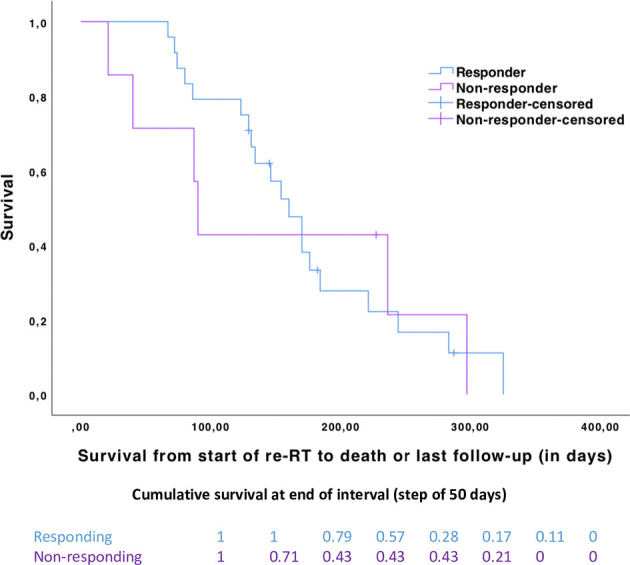
Kaplan–Meier curves with survival from the start of second-line treatment for responding (*n* = 24, in blue) and non-responding (*n* = 7, in purple) patients (log-rank test, *p*-value = 0.871). Cumulative proportion surviving at the end of the interval by a step of 50 days between the two groups.

These analyses revealed a median survival (in days) after the start of re-RT of 150 days (range, 67 to 325 days) in DIPG patients with documented clinical benefit (*n* = 24) versus 90 days (range, 21 to 297 days) (**Figure 3**) in DIPG patients without clinical benefit (*n* = 7) (log-rank test, *p*-value = 0.871).

The cumulative survival (by a step of 50 days) is in favor of responding patients during the first 150 days, and later survival of the two groups converges (**Figure 3**).

### Impact of Patients’ Characteristics on Re-RT Mediated Clinical Benefit

Clinical improvement to re-RT is not impacted by patients’ gender (female responders versus male responders, Fisher’s exact test *p*-value = 1) or age (patients ≤6 years versus >6 years, Fisher’s exact test *p*-value = 0.394).

A total dose <20 Gy was associated to a global clinical response rate of 71% (*n* = 10/14), whereas patients who received ≥20 Gy had clinical benefits in 82% (*n* = 14/17) of the cases (Fisher’s exact test, *p*-value = 0.671). **Table 2** describes neurological triad improvement for 25 patients according to the median re-RT dose given <20 Gy (*n* = 13) or ≥20 Gy (*n* = 12). The distinct regimen of re-RT dose does not provide evidence for differences with regard to CN, LT signs, headaches, and fatigue improvements. However, DIPG patients receiving ≥20 Gy re-RT dose may improve slightly better with regard to ataxia (67% versus 23%, Chi-square *p*-value = 0.028).

**Table 2:** Global clinical benefits following re-irradiation (re-RT) of all patients (*n* = 31; Mann–Whitney *U* test *p*-value = 0.671) and the detailed clinical benefits of well-documented patients (*n* = 25) grouped according to the re-RT dose given (<20 Gy versus ≥20 Gy).

Re-RT dose given (Gy) Clinical benefits		< 20 (*n* = 14)		≥ 20 (*n* = 17)	
**Global clinical benefits (*n* = 31)**					
	Responding	10	71%	14	82%
	Non-responding	4	29%	3	18%
					
		< 20 (*n* = 13)		≥ 20 (*n* = 12)	
**Detailed clinical benefits (*n* = 25)**					
	Cranial nerve	7		4	
	Long tract	6		4	
	Cerebellar	3		8	
	Fatigue	1		5	
	Headaches	2		3	

A difference appears to exist (23% versus 67%, chi-square p-value = 0.028) in improvement of cerebellar signs between the two groups (<20 Gy versus ≥20 Gy).

## Discussion

Our analysis demonstrates that re-RT provides a neurological benefit in two-thirds (*n* = 16/25) of the patients, with an improvement of at least one symptom of the neurological triad. An amelioration in each symptom of the neurological triad (CN, LT, and CB), independently, amounts to approximately 40%, and a re-RT dose ≥20 Gy might have a positive impact on ataxia improvement.

To our knowledge, the spectrum of neurological triad improvement after re-RT has not yet been investigated, thus denying us the possibility for comparison with previous studies. However, Tinkle et al. assessed the evolution of neurological triad symptoms of DIPG patients undergoing upfront RT. At the end of the upfront RT, 87.9% (*n* = 73/83), 85.4% (*n* = 88/103), and 80.8% (*n* = 63/78) of patients had improvement in CB, CN, and LT signs, respectively (2). Focusing on neurological improvement after re-RT, we demonstrated that 44% (*n* = 11/25), 40% (*n* = 10/25), and 44% (*n* = 11/25) of patients had improvement in CN, LT, and CB signs, respectively. Comparison of these two studies is limited by several differences. The study of Tinkle et al. reported on a larger and prospective study about RT-naive DIPG patients, investigating the impact of a median cumulative upfront RT dose of 54 Gy on neurological triad improvement (2).

The same study extensively investigated the influence of neurologic symptom change during RT on survival outcomes (2). Survival was longer in patients who experienced an improvement in cranial nerve deficits, as compared to patients who did not experience such an improvement (2). Because the number of patients in our study was smaller, we only compared the survival time (from re-RT to death), according to the global clinical response to re-RT. Survival was not different and may be partly explained by the small number of responding (*n* = 24) and non-responding (*n* = 7) patients in our study and the relatively short—6 months on average (15)—survival time of progressive DIPG patients treated by re-irradiation.

Of interest, Tinkle et al. evaluated the impact of clinical variables (age, sex, race, cranial spinal fluid before or during RT, concurrent systemic chemotherapy to RT, and clinical target volume) on the cumulative upfront RT dose associated with neurological improvement. Except for race where black patients needed lower RT dose to show improvement in LT signs, a significant correlation between variables and symptom improvement did not exist (2). With our study, we provide evidence that neurological benefits with regard to cerebellar sign or ataxia amelioration are likely to be more frequent in patients receiving a total re-RT dose of ≥20 Gy (*p*-value = 0.028). This appears to be in agreement with the above-mentioned publication reporting on a relative high upfront median RT dose of approximately 21.6 Gy to obtain first cerebellar sign improvement (2). However, among patients who received ≥20 Gy (*n* = 17), only 6 received 30 Gy, while the others received 20 Gy. In the same way, among the <20 Gy (*n* = 14) patient group, only 5 have had an 18-Gy regimen, while the others received 19.8 Gy, which means that, between the two groups, most of the patients (*n* = 20) received a re-RT dose of 19.8 to 20 Gy. Thus, the difference of neurological benefits regarding ataxia may be that the 30 Gy cohort are contributing the benefit demonstrated, but these results obtained by our explorative and hypothesis generating analyses need to be confirmed in larger series. 

The challenges of retrospective analysis have already been discussed in our previous publication (7). However, focusing on the neurological response to re-RT and by splitting the patients into two groups according to their clinical response, we are confronted by small sample sizes and some incomplete data. We especially experienced a lack of information for six patients, partly explained by the fact that data were collected in various centers among Europe, and no common case report form was used to assess the clinical benefits. Based on previous studies, the main limitation is the absence of detailed information concerning the symptomatic status of the patients before re-RT. Indeed, this would allow us to tailor the response rate to re-RT based on the pre-re-RT status of patients since symptoms cannot improve if they do not exist before the onset of re-RT. Referring to the study cited above (2), *n* = 108 patients were considered for the study, *n* = 78 had LT signs at diagnosis before undergoing first RT, and *n* = 63 (80.8%) had improvement in LT signs after RT. In the absence of a detailed pre-RT evaluation, the LT sign improvement rate would have been slightly underestimated (*n* = 63/108; 58.3%). In the same way, our results potentially underestimate the real rate of neurological improvement post re-RT. Even in the literature, the percentages of patients with the classical triad at first progression before undergoing further treatment such as re-RT are not well documented. Many studies—including our article—investigating the impact of re-RT in DIPG indicate that all patients had neurological symptoms at progression (7, 8, 12). In comparison, approximately 100% of patients suffer from one neurological deficit, 85% of patients suffer from two or more neurological deficits, and 57% of patients experience the classical triad before undergoing upfront RT (2). In addition, we were unable to assess the weaning of steroids use—although this represents an important aspect—after re-RT in general or between the responding and non-responding DIPG patients to re-RT. Lassaleta et al. reported that re-RT allowed a weaning of steroids use in 4 out of 9 DIPG patients (44%) who were using steroids at first progression (12). We are unable to exclude the possibility that steroid use was more frequent in one or the other group. Lastly, the molecular profile may potentially influence the clinical response rate in progressive DIPG patients to re-RT, and this should be investigated in biopsied DIPG cohorts (6). In our present study, reporting about DIPG patients re-irradiated between 2011 and 2015, we do not have molecular data of the tumors of the DIPG patients.

Finally, evaluation of quality of life is lacking in our and other retrospective earlier reports (7–10, 12–14). As this information is missing and relevant for families confronted with a decision on how to proceed further with regard to treatment strategies when experiencing progressive disease in their child with a DIPG, quality-of-life data are currently prospectively assessed using the re-irradiation strategy in several trials including the prospective Canadian protocol (17).

## Conclusion

This analysis demonstrates that among 25 patients with well-documented clinical benefits, a median re-irradiation dose of 20.0 Gy results in an improvement of at least one symptom of the neurological triad in two-thirds of DIPG patients. An improvement in each symptom of the neurological triad (CN, LT, and CB), independently, amounts to approximately 40%. No difference in survival after the start of re-RT was observed between the responding and non-responding patients in this limited cohort. DIPG patients receiving ≥20 Gy appear to improve slightly better with regard to ataxia; however, we need more data to verify whether higher doses of approximately 30 Gy might provide additional clinical benefits. To improve the strength of such a study, we need to increase the number of patients and ideally perform a prospective study.

## Data Availability Statement

The original contributions presented in the study are included in the article/supplementary material. Further inquiries can be directed to the corresponding author.

## Ethics Statement

The studies involving human participants were reviewed and approved by Commission cantonale d’éthique de la recherche CCER (Geneva), confirming (Req-2022-00407) that this project does not need to be reviewed by our ethics committee, the Geneva CCER, according to Swiss law. The Human Research Act (HRA) applies to research concerning human diseases and concerning the structure and function of the human body (as defined in the Art 2). Written informed consent to participate in this study was provided by the participants’ legal guardian/next of kin.

## Author Contributions

AB conceived the idea of the study and the principal study design. GJ, LC, CK, and AB contributed to the concept and design of the study. GJ, LG, SB, HM, MR-A, KB, HB, BH, AM, R-DK, DH, CS, MM, EP, VB, AB, DV, and CK were responsible for the acquisition of data. Quality control of data and algorithms was done by GJ, LC, and AB. Statistical analysis was done by LC and AB. GJ, LC, and AB participated in the analysis and interpretation of the data. Manuscript preparation and editing was done by LC, AB, and GJ, respectively. All authors contributed to the article and approved the submitted version.

## Funding

This work was supported in part by Deutsche Kinderkrebsstiftung. Open access funding was provided by the University Of Geneva.

## Conflict of Interest

HM was supported by the National Institute of Health Research/Biomedical Research Centre at The Royal Marsden NHS Foundation Trust, Sutton.

DH was supported by the National Institute for Health Research/Biomedical Research Centre at Great Ormond Street Hospital for Children, NHS Foundation Trust, and University College London.

The remaining authors declare that the research was conducted in the absence of any commercial or financial relationships that could be construed as a potential conflict of interest.

## Publisher’s Note

All claims expressed in this article are solely those of the authors and do not necessarily represent those of their affiliated organizations, or those of the publisher, the editors and the reviewers. Any product that may be evaluated in this article, or claim that may be made by its manufacturer, is not guaranteed or endorsed by the publisher.
