# Erratum: Neurological symptom improvement after re-irradiation in patients with diffuse intrinsic pontine glioma: A retrospective analysis of the SIOP-E-HGG/DIPG project

**DOI:** 10.3389/fonc.2023.1182994

**Published:** 2023-03-30

**Authors:** Frontiers Production Office

**Affiliations:** Frontiers Media SA, Lausanne, Switzerland

**Keywords:** diffuse intrinsic pontine glioma (DIPG), radiotherapy, re-irradiation (re-RT), child, adolescent

An omission to the funding section of the original article was made in error. The following sentence has been added: “Open access funding was provided by the University Of Geneva”.

The original version of this article has been updated.

